# Armc8 is an evolutionarily conserved armadillo protein involved in cell–cell adhesion complexes through multiple molecular interactions

**DOI:** 10.1042/BSR20180604

**Published:** 2019-08-02

**Authors:** Ismail Sahin Gul, Paco Hulpiau, Ellen Sanders, Frans van Roy, Jolanda van Hengel

**Affiliations:** 1 Center for Inflammation Research, VIB, Ghent, Belgium; 2 Department of Biomedical Molecular Biology, Ghent University, Ghent, Belgium; 3 Howest, University College West Flanders, Bruges, Belgium; 4 Department of Basic Medical Sciences, Ghent University, Ghent, Belgium

**Keywords:** Armadillo protein, Armc8, desmosome, molecular evolution

## Abstract

Armadillo-repeat-containing protein 8 (Armc8) belongs to the family of armadillo-repeat containing proteins, which have been found to be involved in diverse cellular functions including cell–cell contacts and intracellular signaling. By comparative analyses of armadillo repeat protein structures and genomes from various premetazoan and metazoan species, we identified orthologs of human Armc8 and analyzed in detail the evolutionary relationship of *Armc8* genes and their encoded proteins. Armc8 is a highly ancestral armadillo protein although not present in yeast. Consequently, Armc8 is not the human ortholog of yeast Gid5/Vid28.

Further, we performed a candidate approach to characterize new protein interactors of Armc8. Interactions between Armc8 and specific δ-catenins (plakophilins-1, -2, -3 and p0071) were observed by the yeast two-hybrid approach and confirmed by co-immunoprecipitation and co-localization. We also showed that Armc8 interacts specifically with αE-catenin but neither with αN-catenin nor with αT-catenin. Degradation of αE-catenin has been reported to be important in cancer and to be regulated by Armc8. A similar process may occur with respect to plakophilins in desmosomes. Deregulation of desmosomal proteins has been considered to contribute to tumorigenesis.

## Introduction

The armadillo (Arm) superfamily members are involved in various cellular functions, such as cell–cell adhesion, intracellular transport, signaling and ciliogenesis (reviewed in [1]). Our recent comprehensive bioinformatics study of the armadillo repeat protein family revealed that for instance the human genome encodes at least 70 armadillo proteins [2]. In contrast with the armadillo catenins, the importin-α proteins and the armadillo formins, the functions of other armadillo proteins (40 out of 70, [2]) have not received significant attention. Among the latter is metazoan armadillo-repeat-containing protein 8 (Armc8). Nucleotide sequencing revealed that human Armc8α (longest) and Armc8β proteins are encoded by alternatively spliced products from the same gene, *ARMC8* [3]. The first 364 amino acids (AA) of these isoforms are identical but the Armc8β open reading frame is terminated by an early stop codon and encodes a protein of 385 AA. Armc8α possesses a first armadillo domain comprising four armadillo repeats and a second armadillo domain with five armadillo repeats, which are connected with each other by a large insert or loop region composed of approximately 150 AA. Armc8β lacks the second armadillo domain.

Currently, several studies suggested that in the yeast *Saccharomyces cerevisiae, ARMC8* is homologous to *GID5* (Glucose induced degradation deficient complex subunit 5) or *VID28* (Vacuolar Import and Degradation 28) [3–5]. The *Saccharomyces cerevisiae* GID complex is a large assembly of seven proteins and regulates the proteolysis of fructose-1,6-bisphosphatase (FBPase), a rate-controlling enzyme of gluconeogenesis [6], by polyubiquitination [4]. An alternative mechanism for the degradation of FBPase is the VID pathway in yeast [7]. It has been proposed that the LisH/CTLH (C-terminal to Lissencephaly type-1-like homology (LisH) motif) complex is a metazoan homolog of the *Saccharomyces cerevisiae* GID and VID complexes [3]. The metazoan orthologs of the yeast GID complex members Gid1, Gid4 and Gid5 have been proposed to be, respectively, RanBP9, C17orf39 (official gene symbol *GID4*) and Armc8 in the metazoan LisH/CTLH complex. Compared with the other LisH/CTLH complex members, C17orf39 and Armc8 do not possess LisH/CTLH motifs. It has been claimed that the N-terminal region present in both Armc8α and Armc8β might be important for integration in the LisH/CTLH complex [3]. In the present study we analyzed the structure, intermolecular interactions and evolutionary relationships of Armc8 at a higher level than was previously possible.

Human Armc8 has been also associated with the degradation of αE-catenin, one of the key components of the E-cadherin/β-catenin/α-catenin (CCC) complex [8]. Recent studies suggest that Armc8 is involved in the degradation of E-cadherin and associated catenins in malignant cancers [9]. Catenins can be divided into three subfamilies: β-catenins, δ-catenins and α-catenins. While β-catenin and δ-catenin members possess armadillo repeats, α-catenins have vinculin homology domains [10]. Furthermore, the α-catenin gene family consists of three paralogs: *CTNNA1, -2* and *-3*, encoding αE-, αN- and αT-catenins, respectively, where E stands for epithelial, N for neural and T for testis [11]. All three paralogs are able to interact with β-catenin [11–13] and localize at intercellular junctions [14]. Moreover, it has been shown that in intercalated discs of the heart, αT-catenin can link classical cadherin–catenin complexes to desmosomal cadherins through binding to plakophilins [15]. Most cell–cell adhesion junctions consist of multiprotein complexes, comprising cadherins, catenins, armadillo proteins and close relatives thereof. These components are often emerging as versatile scaffolds for multiple signaling processes that not only facilitate junction dynamics but also more globally regulate diverse cellular activities. Besides assembling typical adhesion junctions, components of desmosomes and adherens junctions may intermingle and form hybrid junctions. With all the different reported armadillo protein interactions in mind, we decided to perform a candidate approach to identify protein interactors of Armc8 rather than to screen for completely novel interactors. We thus identified Armc8 as an interaction partner of specific δ-catenin members and of αE-catenin.

## Materials and methods

### Sequence searches and phylogenetic analyses

To identify the orthologs of Armc8 and Armc8-like proteins, the following metazoan and non-metazoan proteomes were investigated by BLASTp searches. Non-metazoans were represented by *Capsaspora owczarzaki, Monosiga brevicollis* and *Salpingoeca rosetta*. The non-bilaterian metazoans were represented by *Nematostella vectensis, Trichoplax adhaerens, Amphimedon queenslandica and Mnemiopsis leidyi*. Finally, we investigated six bilaterian species: *Drosophila melanogaster, Aplysia californica, Ciona intestinalis, Callorhinchus milii, Danio rerio, Gallus gallus, Mus musculus* and *Homo sapiens*. The sequence with the highest homology (lowest *E*-value) was considered as ‘best-hit’. To confirm the potential orthologous relations of these best hits, we performed reciprocal best-hit analysis (rBLASTp). All the obtained best hits from the previous step were retrieved and used as a query for BLASTp searches against the human proteome.

Additionally, to investigate ARM protein orthologs in yeast *Saccharomyces cerevisiae*, a search with the ARM repeat hidden markov model PF00514 from Pfam was performed against the proteome of *Saccharomyces cerevisiae*. We also looked for putative armadillo yeast proteins in the NCBI Gene database and the Superfamily (supfam.org) database.

For multisequence homology analysis, armadillo repeat regions of all investigated protein sequences were aligned with ClustalX. Phylogenetic trees were constructed with two methods: neighbor-joining in ClustalX (with bootstrap) and Bayesian analysis in Mr Bayes. The phylogenetic trees were visualized with the Interactive Tree Of Life (iTOL) tool [16].

### Construction of expression plasmids

*Homo sapiens* Armc8α cDNA (clone MGC: 48800 IMAGE: 5240423, complete coding sequence) and *Homo sapiens* Armc8β cDNA (clone MGC: 10058 IMAGE: 3892143, complete coding sequence) in pCMV_SPORT6 backbone were purchased from ImaGenes Source Bioscience. Using these plasmids as templates, full-length Armc8α and Armc8β cDNA were PCR amplified with gene-specific primers (Supplementary Table S1), containing the AttB sites and stop codons for subsequent Gateway cloning (Invitrogen, Carlsbad, CA, U.S.A.). Amplified fragments were precipitated and inserted into pDONR207 (Invitrogen) by the BP recombination reaction, yielding pDONR207_Armc8α and pDONR207_Armc8β. C-terminal Armc8α (C-term, encoding AA 268-659) and Armc8α 2nd Arm (encoding AA 352-659) were amplified with gene-specific primers containing the AttB sites (Supplementary Table S1), using the pDONR207_Armc8α template. The obtained fragments were inserted into pDONR207 (Invitrogen) by the BP recombination. The C-terminally truncated Armc8β (encoding AA 268-385) was amplified using pDONR207_Armc8β template with gene-specific primers containing the AttB sites (Supplementary Table S1) and inserted into pDONR207 (Invitrogen) by the BP recombination. LR reaction between the entry clones (Supplementary Table S1) and the destination vectors, pGADT7 and pGBKT7, for yeast two-hybrid (Y2H) experiments, and pdcDNA-FLAG for cell culture experiments, produced the expression plasmids listed in Supplementary Table S2 [15,17–20].

### Yeast two-hybrid assays

The Matchmaker Gold Y2H system (Clontech) was used with the Y2H Gold yeast strain for protein interaction tests as described previously [21]. Briefly, 1 μg of each bait and prey plasmid were co-transformed using the lithium acetate/single-stranded carrier DNA method and transformed yeasts were then plated on minimal synthetic drop-out medium (SD) lacking leucine and tryptophan (SD–LW). After 3–5 days, colonies were picked and grown overnight in the SD–LW medium. Replica plates selecting for prey–bait interactions in transformed yeasts were made in SD medium lacking leucine, tryptophan, histidine and alanine (SD–LWHA), but containing 40 μg/ml 5-bromo-4-chloro-3-indolyl-β-d-galactopyranoside. Experiments were performed in triplicate and repeated three times.

### Cell lines and stainings

The SKCO-15 cell line was cultured in Dulbecco’s modified Eagle’s medium (DMEM) supplemented with 10% fetal calf serum, penicillin (100 IU/ml), streptomycin (0.1 mg/ml), L-glutamine (0.03%) and 15 mM HEPES (4-(2-hydroxyethyl)-1-piperazineethanesulfonic acid). Cells were grown in a humidified incubator at 37°C and 5% CO_2_.

For immunofluorescence experiments, SKCO-15 cells were seeded on coverslips in six-well plates. Forty-eight hours post transfection, cells were washed with phosphate buffered saline (PBS) and fixed in 100% methanol for 20 min at −20°C. After washing the wells with PBS, cells were immunostained with primary antibodies (rat monoclonal anti-PKP3 ([22]), mouse monoclonal anti-Armc8 antibody (WH0025852M1, Sigma) or rabbit polyclonal anti-Armc8 antibody (SAB1401607, Sigma) for 2 h at room temperature (RT). After three washing steps with PBS, cells were incubated with secondary antibodies (diluted in PBS containing 2% gelatin) for 1 h at RT. After several washing steps in PBS, cells were counterstained with Vectashield mounting medium containing DAPI (Vector laboratories Ltd.). Images were taken on a TCS SP5 AOBS confocal microscope (Leica) or on a CellM fluorescence microscope (Olympus).

### Immunoprecipitation (IP) and immunoblot analysis

SKCO-15 cells were lysed in IP lysis buffer (10 mM Tris-HCL, pH7.4, 150 mM NaCl and 0.5% NP40 and Roche complete protease inhibitor cocktail mix). Whole protein lysates and precipitates were analyzed by Western blotting. Proteins were separated by SDS-PAGE and then transferred to PVDF membranes (Millipore). After blocking with 5% non-fat dry milk in PBS containing 0.1% Tween-20, membranes were incubated with primary antibodies (see above) for 2 h at RT. After several washing steps in PBS, membranes were incubated for 1 h with secondary horseradish peroxidase (HRP)-conjugated antibodies. Detection was performed using the ECL detection system (Amersham GE healthcare).

## Results

### Identification of Armc8 orthologs and their phylogenetic relationships

To identify the orthologs of the human Armc8, we searched the proteomes of 15 metazoan and non-metazoan species with (reciprocal) rBLASTp. Sequence searches revealed that except for the *Drosophila melanogaster* (fruit fly) genome, all investigated metazoan species encode Armc8 in their genomes (Table 1). Armc8 is absent in the non-metazoan choanoflagellates, *Monosiga brevicollis* and *Salpingoeca rosetta*. However, the genome of the single-celled eukaryote *Capsaspora owczarzaki* encodes a putative ancestral Armc8 protein (named CAOG_05895) (Table 1). We could not detect any paralogs of the Armc8 protein in any investigated metazoan and non-metazoan species and therefore Armc8 can be considered to be directly related to other armadillo proteins calcium-binding protein 39 (Cab39) and Uso1 (also known as general vesicular transport factor, p115), while being much less similar to Armc2, Armc3, Armc4 and Armc6 (See Figure 3 and ‘Results’ section in [2]).

**Table 1:** Reciprocal BLASTp searches of Armc8 proteins to identify orthologous sequences in metazoans and non-metazoans

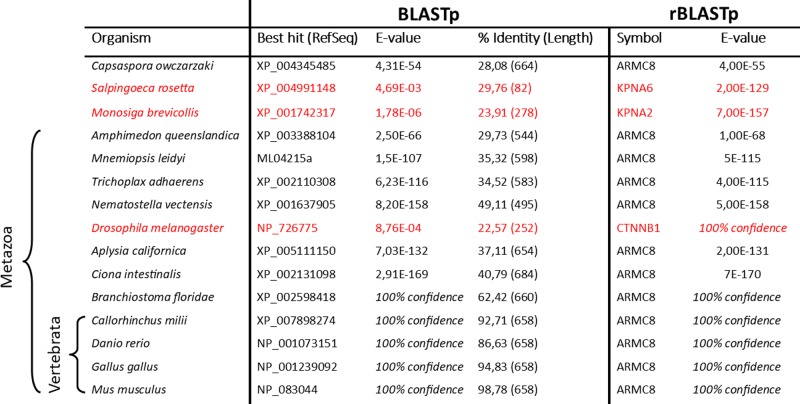

BLASTp searches (left) of the validated human ARMC8α protein against the proteomes of deuterostomes *Ciona intestinalis* (vase tunicate), *Branchiostoma floridae* (Florida lancelet), *Callorhinchus milii* (elephant shark), *Danio rerio* (zebrafish), *Gallus gallus* (chicken) and *Mus musculus* (mouse) of the protostomes *Aplysia californica* (sea hare) and *Drosophila melanogaster* (fruit fly), of the non-bilaterians *Nematostella vectensis* (sea anemone), *Trichoplax adhaerens* (placozoan), *Amphimedon queenslandica* (sponge), *Mnemiopsis leidyi* (comb jelly), of the unicellular choanoflagellates (*Monosiga brevicollis* and *Salpingoeca rosetta*, close relatives of metazoans) and of the filasterean lineage (*Capsaspora owczarzaki*). The reciprocal BLASTp results (right) confirmed the orthology of the best-hits (black text) or not (red text). The table provides alignment length and identity values.

To investigate the potential orthologous relationship of Armc8 with the yeast Gid/Vid complex members and mammalian Lish/CTLH complex members, we searched the Gid/Vid members in the human proteome by BLASTp. Results of this search suggested that the yeast Gid1, -2, -4, -7 and -9 proteins show strong similarity to the human Ranbp10, Rmnd5a, Gid4, Wdr26 and Maea proteins, respectively (Supplementary Table S3). However, searching the yeast Gid5 ortholog in the human proteome identified Tspyl2 (testis-specific Y-encoded-like protein 2) and not Armc8 (Supplementary Table S3). Moreover, searching the yeast proteome with any of the identified Armc8 orthologs (Table 1) did not identify the yeast Gid5/Vid28 protein sequence either (Supplementary Table S4); instead, Kap123p (Karyopherin-α) was identified as best hit.

In an alternative approach, we identified all armadillo proteins in yeast by performing a HMM search with the pfam model PF00514 against the proteome of Saccharomyces. We also looked for putative armadillo yeast proteins in NCBI Gene and Superfamily (supfam.org) databases. In total, 12 putative armadillo proteins were found (table ARM-yeast-proteins, Supplementary Table S5). Only five of these have annotated armadillo repeats, the other seven have HEAT repeats instead. Remarkably, yeast Vid28 was not among these.

Nevertheless, Vid28 was included in a phylogenetic analysis including the 12 armadillo proteins in yeast, several known human armadillo proteins, and known orthologs in the single-celled eukaryote *Capsaspora owczarzaki* which is one of the closest unicellular relatives to animals [2]. Two different methods were used: neighbor-joining (with bootstrap) and Bayesian analysis. Both phylogenetic trees (Supplementary Figure S1) confirmed all human (Hs) and *Capsaspora owczarzaki* (Co) orthologs, but only for yeast (Sc) Kpna1/Srp1 support is found as being an ortholog of human and *Capsaspora owczarzaki* Kpna1 proteins. Clearly, yeast Vid28 is not an ortholog of Armc8. In line with this, yeast Vid28 contains only a few HEAT repeats but no ARM repeats.

It is also noteworthy that, although a PSI-BLAST of human Armc8 finds yeast Vid28 as a hit, only a short region aligns with not more than 21% AA sequence identity. Moreover, this local alignment shows several gaps most probably caused by the difference between ARM and HEAT repeats. A single HEAT repeat contains only two α helices compared with three helices in one ARM repeat. HEAT repeats form a superhelical 3D structure comparable but not identical to that of ARM repeats, and it has been suggested that HEAT and ARM repeat proteins are evolutionary related [2,23]. Collectively, these data indicates that the yeast Gid5/Vid28 is not the ortholog of Armc8 and that a genuine ortholog of metazoan Armc8 is not encoded by the yeast genome.

**Figure 1:**
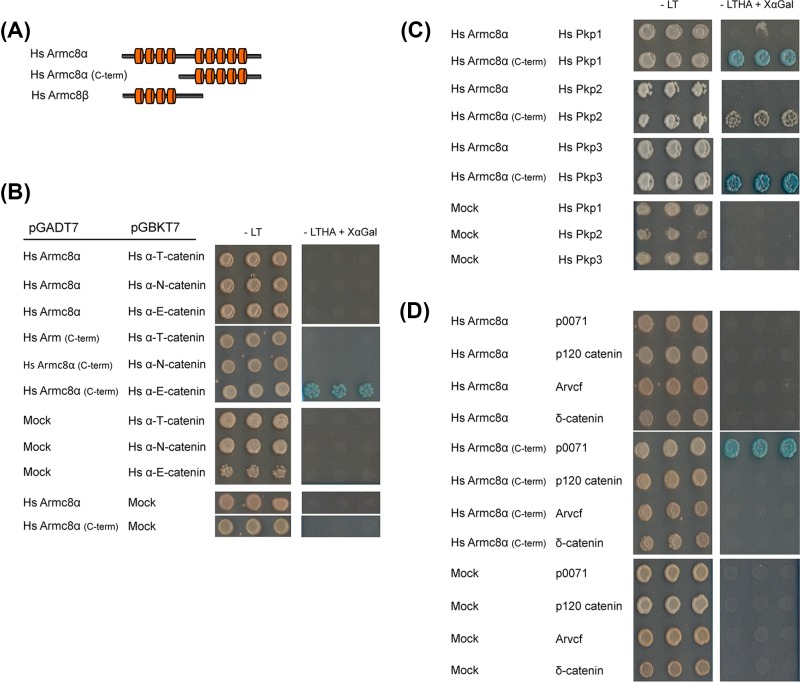
Yeast two-hybrid (Y2H) assay to study the possible interactions between human Armc8α and members of the α- and δ-catenin protein families (**A**) Schematic representation of the Armc8 protein constructs used. Armadillo repeats are represented by red cylinders. (**B**–**D**) The Matchmaker Gold yeast strain Y2H Gold was co-transformed with different expression plasmids. All transformations shown were successful since transformed yeasts grew on SD-LT (L = leucine; T = tryptophan) selective medium (left panels, -LT). Data are each time shown as three representative yeast colonies. The Y2H Gold strain allows reliable detection of one-to-one protein interactions as the rescued GAL4 expression activates the selection and reporter genes ADE2, HIS3 and MEL1 (α-galactosidase). Positive interactions yield blue colonies on SD–LTHA (H = histidine; A = adenine) containing X-α-Galactosidase (right panels, -LTHA + XαGal). All results were reproduced in at least three biological replicates. For all bait and prey vectors used, a test for auto-activation was negative. **(B)** Testing the interaction between full-length human Armc8α or a C-terminal Armc8α fragment against the three α-catenin paralogs identified only αE-catenin as interaction partner of Armc8α (C-term). **(C)** Testing the interaction between the two Armc8α constructs against plakophilins showed that the Armc8α (C-term) could interact with all plakophilins. **(D)** Testing the interaction between the two Armc8α constructs against the CTNND core proteins identified p0071 but not the other armadillo proteins as interaction partner of Armc8α (C-term).

**Figure 2:**
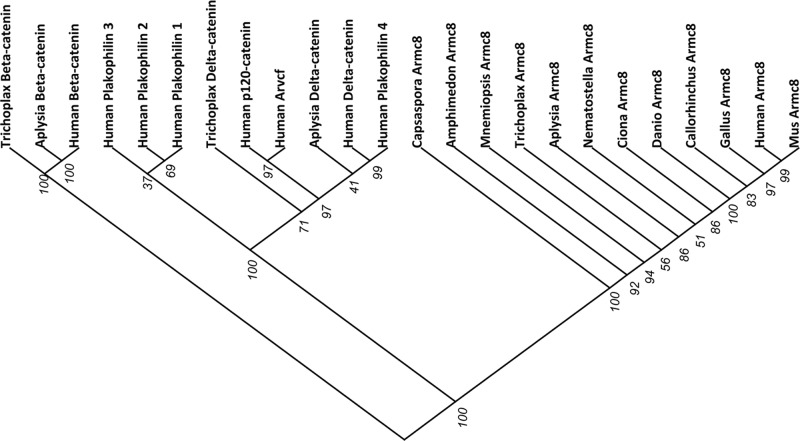
Phylogenetic analysis of identified Armc8 orthologs and of armadillo catenins Protein sequences of human Armc8α and its orthologs in metazoan and non-metazoan species (see Table 1) were aligned mutually and with human, California sea hare (*Aplysia californica*) and placozoan (*Trichoplax adhaerens*) armadillo catenin proteins. The phylogenetic analysis was performed with the neighbor-joining method and bootstrap values were provided for each branch. The phylogenetic tree was visualized using Dendroscope [40].

Similar to the δ-catenin members, human Armc8α contains nine ARM repeats interrupted by a long insert between ARM4 and -5 [24] (Figure 1A). Several δ-catenin members [15] and also Armc8 have been shown to interact with α-catenin [8,15]. Despite this correlation in structure and function, the phylogenetic relationship between Armc8 and armadillo catenins (β- and δ-catenins) is not clear. In addition to the identified Armc8 orthologs listed in Table 1, we included the protein sequences of armadillo catenins to investigate any mutual relationships. Neighbor-joining phylogeny clades the *Capsaspora owczarzaki* CAOG_05895 and *Amphimedon queenslandica* Armc8-like proteins together with the other identified metazoan Armc8 proteins, and this with very high bootstrap (BS) values of 100 and 92, respectively (Figure 2). On the other hand, δ- and β-catenins formed clades clearly separated from the Armc8 sequences and from each other, resulting in three monophyletic groups with BS values of 100 (Figure 2).

### Conservation and syntenic relationship of chordate ARMC8 genes

**Figure 3:**
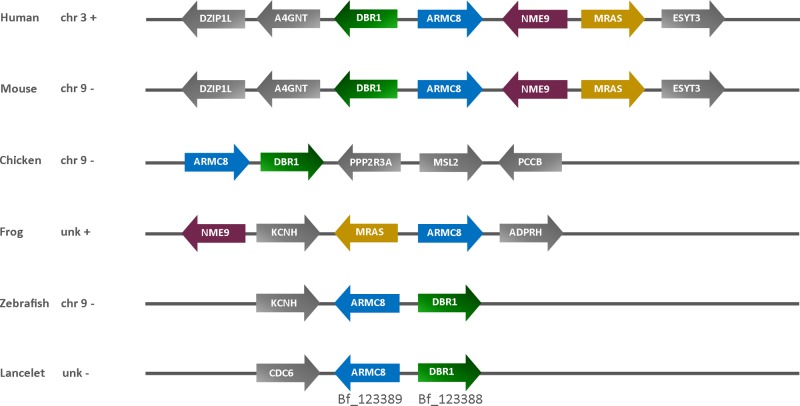
Synteny of *ARMC8* and flanking genes in chordates Comparison of the syntenic blocks shared between human chromosome 3q22.3 and corresponding chromosomal regions in four other vertebrates (*Mus musculus, Gallus gallus, Xenopus tropicalis and Danio rerio*) and the chordate *Branchiostoma floridae*. In each genomic region analyzed, *ARMC8* is represented by a blue arrow.

To examine the level of synteny among the identified orthologs of *ARMC8* genes, we compared its location on human chromosome 3q22.3 with corresponding chromosomal regions in several vertebrate species. Human *ARMC8* is located downstream of *DBR1* (encoding Debranching RNA Lariats 1) and upstream of *NME9* (encoding Thioredoxin domain-containing protein 6) (*NME9*) and *MRAS* (encoding Muscle RAS Oncogene Homolog) (Figure 3). The last exon of *NME9* is partially nested in the *ARMC8* gene. Comparison of this chromosomal organization with the *ARMC8* genes from other vertebrates revealed that at least one of the flanking human genes was also in close proximity of *ARMC8* in the other genomes (Figure 3). In lancelet *Branchiostoma floridae, ARMC8* has been annotated as Bf_123389 with flanking gene *DBR1* (Bf_123388). The conserved synteny of the *ARMC8* neighboring genes in chordates further indicates that *ARMC8* is an evolutionarily conserved gene.

### Armc8 is interacting specifically with αE-catenin and with plakophihlins

**Figure 4:**
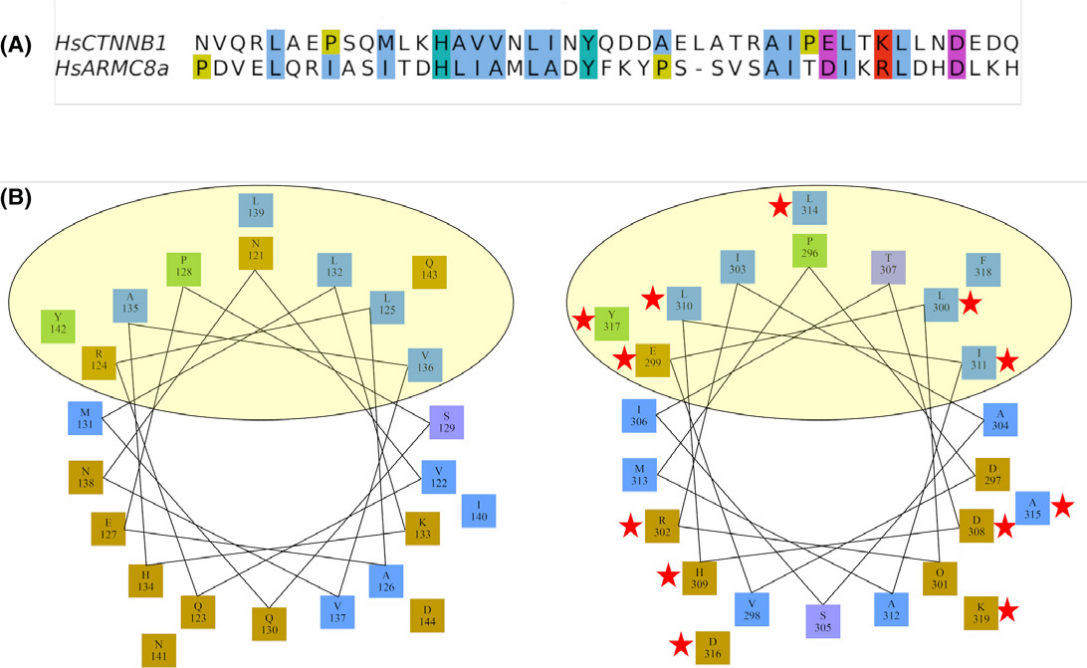
Alignment and helical wheel representation of the α-catenin binding regions in human β-catenin and Armc8α (**A**) Alignment of human β-catenin region AA 121–165 and human Armc8α region AA 296–339 shows the sequence similarity of both α-catenin binding regions. (**B**) Left, characterized binding helices in human β-catenin. The yellow ellipse highlights the evolutionarily conserved surface that binds αE-catenin [25]. Right, predicted α-catenin binding region in human Armc8α; the yellow ellipse highlights the evolutionarily conserved putative surface binding αE-catenin. Similar AA in the predicted α-catenin binding regions are indicated with red stars. Detailed results of the prediction to support this are included as Supplementary Figure S2.

In order to confirm and analyze the possible interactions between human Armc8α, which is the longest isoform in man (Figure 1A), and the various α-catenin paralogs, we performed yeast-two hybrid (Y2H) experiments. The results show that a C-terminal construct of Armc8α (AA 268–659, lacking the first armadillo domain) was able to interact with αE-catenin but neither with αT-catenin nor with αN-catenin (Figure 1B). Remarkably, the full-length Armc8α did not bind any of the α-catenin family members (Figure 1B). Interestingly, we found a significant similarity between the α-catenin binding region of β-catenin [25] with a region of Armc8α predicted by us to be α-catenin binding (Figure 4) and Supplementary Figure S2.

We then investigated the possible role of Armc8 in the desmosomal context. We have proposed that delta-catenins can be divided into two branches: the plakophilin branch and the p120ctn branch (CTNND core proteins) [24]. Both branches comprise desmosomal components. Remarkably, the partial construct of Armc8α was able to interact in the Y2H system with all plakophilins (encoded by *PKP1* to *-3* in man) (Figure 1C). Then, we tested the full-length and the partial Armc8α against the human CTNND core protein family members what revealed that only p0071 (also known as plakophilin-4, encoded by *PKP4* in man) could interact with the partial Armc8α (Figure 1D). Here also the full-length Armc8α did not interact in the Y2H assay with any of the CTNND core proteins (Figure 1D).

### Further characterization of the interaction between Armc8 and other armadillo proteins

**Figure 5:**
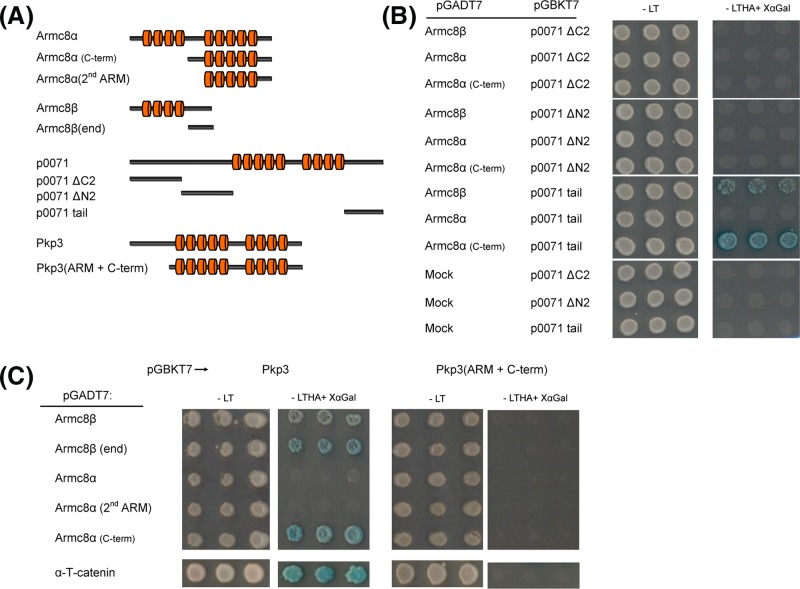
Y2H assay to narrow down the interactions between Armc8, Pkp3 and p0071 proteins (**A**) Schematic structures of Armc8α, Armc8β, Pkp3, p0071 and their truncated forms used in this experiment. Armadillo repeats are represented by red cylinders. (**B**) Testing the interaction between Armc8α and Armc8β constructs against the N- and C-terminally truncated p0071 proteins characterized the tail region of p0071 as binding region to both Armc8α (C-term) and Armc8β. (**C**) Testing the interaction between Armc8α and Armc8β constructs against the full-length Pkp3 and a truncated derivative suggests that the N-terminal domain of Pkp3 is required for the interaction with a region shared by Armc8α and Armc8β.

To narrow down the respective Armc8 and plakophilin domains responsible for the mutual interactions between these proteins, we included the short human Armc8 isoform (Hs Armc8β) in Y2H experiments and also generated a series of human truncation mutants of Armc8α, Armc8β, Pkp3 and p0071 (Figure 5A). Using different C- and N- terminally truncated constructs of p0071, we found that the fragment containing the tail region of p0071 could interact with both Armc8β and the C-terminal fragment of Armc8α (Figure 5B). C-terminally truncated head domains of p0071, named ΔN2 and ΔC2, could not bind to any of the Armc8 constructs (Figure 5B). Next, we tested the interaction between Armc8β and Armc8α against the full-length and N-terminally truncated (ARM + C-terminal) of Pkp3. We observed an interaction of the full-length Pkp3 with both Armc8β and the Armc8α C-term construct (Figure 5C). However, the Pkp3 (ARM + C-terminal) did not interact with either Armc8β or Armc8α (Figure 5C). An additional N-terminally truncated Armc8β, called Armc8β (end) (AA 268–385) and lacking the first ARM domain of Armc8β, was also able to interact with the full-length Pkp3 (Figure 5C). Collectively, this indicates that a shared region of Armc8α and Armc8β could interact with the N-terminal region of Pkp3. To confirm this, we deleted this shared region from Armc8α leading to a construct, called Armc8α (2nd ARM) (AA 352–659) (Figure 5A). As predicted, Armc8α (2nd ARM) did bind neither the full-length Pkp3 nor the Pkp3 (ARM + C-term) (Figure 5C).

### Association of endogenous Armc8 and Pkp3 in SKCO-15 cells

**Figure 6:**
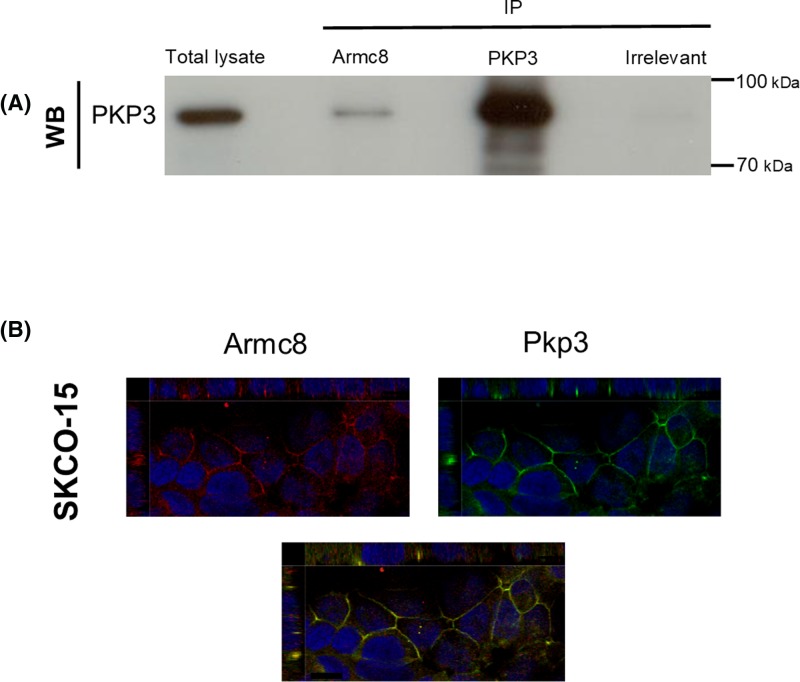
Association of endogenous Armc8α and Pkp3 in SKCO-15 cells (**A**) Endogenous Pkp3 was co-immunoprecipitated with Armc8 from a SKCO-15 cell lysate. The specificity of the antibodies used is as indicated. Initial immunoprecipation (IP) was followed by detection of Pkp3 in a Western blot (WB) of the immunoprecipitates. No interaction was observed in the control immunoprecipitation with an anti-Flag antibody. (**B**) Orthographic and planar projections were obtained by confocal microscopy. Images of SKCO-15 cells showing co-localiation of endogenous Armc8 (red) and Pkp3 (green) at cell–cell contacts. In the lower panel, regions of co-localization appear as yellow. Specific monoclonal antibodies were used; scale bars = 10 µm.

A molecular interaction between Armc8 with Pkp3 was confirmed by co-immunoprecipitation of the endogenous proteins from human colorectal adenocarcinoma cells SKCO-15 (Figure 6A). Intracellular distributions of Pkp3 and Armc8 were investigated through immunofluorescent microscopy of SKCO-15 cells (Figure 6B). Endogenous Armc8 and Pkp3 co-localized both in the cytoplasm and at cell–cell contacts. The localization of Armc8 at cell–cell contacts was also seen with a second independent antibody (rabbit poyclonal antibody, data not shown).

## Discussion

Human armadillo-repeat protein Armc8 was originally identified as a component of the LisH/CTLH complex, a putative ortholog of the yeast GID and VID complexes, which regulates the degradation of FBPase [3]. However, functional studies in Armc8-knockdown mouse C2C12 cell derivatives showed that Armc8α and Armc8β were not essential for the formation of the LisH/CTLH complex [8]. Our BLAST searches of the yeast Gid5/Vid28 against the human genome identified Tspyl2 as best hit (with an *E*-value of 0.83) (Supplementary Table S3). We used multiple identified metazoan Armc8 orthologs (Table 1) for searching the yeast genome by BLASTp and identified Karyopherin-β (Kap123p) as best hit but not the yeast Gid5/Vid28 protein (Supplementary Table S4). Collectively, this suggests that metazoan Armc8 does not show any sequence or functional similarity with the yeast Gid5/Vid28 protein. We therefore propose that the suggested aliases and synonyms proposed in NCBI Gene Entrez, HGNC and GeneCards databases for human ARMC8 (i.e. GID5 and VID28) should be abandoned.

The human *ARMC8* gene is located on chromosome 3 and flanked by *DBR1, NME9* and *MRAS* genes. We were able to detect one or more of those flanking genes in the close vicinity of *ARMC8* genes from mouse, chicken, frog and lancelet *Branchiostoma floridae* (Figure 3). Together with the pairwise sequence searches, this conserved synteny indicates that the human *ARMC8* gene is indeed orthologous to other identified *Armc8* genes.

It has been shown that Armc8 is involved in the proteasome-dependent degradation of αE-catenin [8]. Together with β-catenin and the cytoplasmic tail of classical cadherins, αE-catenin forms the CCC complex mediating the junctional coupling of neighboring cells and the dynamic anchoring to the underlying actin cytoskeleton [26]. Reduced or altered expression of the CCC complex members is found in various human cancers [27]. In contrast with adherens junctions comprising classical cadherins and catenins in a CCC, desmosomal cadherins and their associated plakophilins mediate adhesion at desmosomes. Desmosomes are macromolecular junctions that tether intermediate filaments to the plasma membrane and are responsible for mediating strong cell–cell adhesion in for instance epidermis and myocardium.

Lately, several studies postulated different effects of Armc8 on the CCC in human cancers. Jiang et al. [28] reported that Armc8 can modify the CCC by regulating the expression levels of metalloproteinase-7 and Snail, which is a mediator of E-cadherin repression. Armc8 was reported to contribute to malignancy in ovarian [29], lung [30,31], colon [28], osteosarcoma [32] and breast cancers [33]. Overexpression of Armc8 in the ovary adenocarcinoma SK-OV-3 cells led to increased expression of Snail [34], and to decreased expression of CCC members [28]. Knockdown of Armc8 in the hepatocellular carcinoma HepG2 cell line significantly up-regulated the expression levels of E-cadherin, β-catenin and αE-catenin [9]. Silencing of Armc8 inhibited TGF-β-induced epithelial–mesenchymal transduction in bladder carcinoma UMUC3 cells [35]. These findings emphasize that Armc8 is negatively involved in the regulation of the CCC complex.

In the present study, we showed in the Y2H assay that a C-terminal domain of human Armc8α is able to interact with human αE-catenin but neither with αN-catenin nor with αT-catenin (Figure 1B). Interestingly, we found a putative binding region in Armc8α, which resembles the structurally characterized α-catenin binding region of β-catenin [25] (Figure 4). In full-length β-catenin, the α-catenin binding region is N-terminally adjacent to the central 12 armadillo repeats [25]. In contrast, we found that the putative α-catenin binding region of Armc8α resides between its two armadillo domains.

Y2H screening of Armc8 constructs against the delta-catenins identified all plakophilins (Pkp1 to -3) and p0071 (also known as Pkp-4) as being interaction partners of Armc8 (Figures 1 and 5). While the full-length Armc8α was not able to interact in this assay with either plakophilins or p0071, a fragment of Armc8α that lacks the first four armadillo repeats was able to bind both types of partners. Further characterization of these interactions revealed that a region shared between Armc8α and Armc8β was sufficient to bind the N-terminal of Pkp3 and the tail of p0071 (Figure 5). Immunofluorescence experiments on epithelial tumor cell line SKCO-15 showed that in this cell type the endogenous Armc8 and Pkp3 co-localized in the cell junctions (Figure 6B). Finally, we used co-immunoprecipitation to show an interaction between endogenous Armc8 and Pkp3 in SKCO-15 cells (Figure 6A).

Tandemly arrayed armadillo repeats fold into a superhelical structural domain, which enables these repeats to interact with various ligands. For instance, the armadillo domain of β-catenin has been shown to interact with many proteins, including E-cadherin, Axin and Tcf, and to be involved in molecular complexes with functions in adherens junctions, specific protein degradation and Wnt signaling (reviewed in [36]). Yet the physiological interaction partner(s) of the armadillo domains of Armc8 are to be reported, and its possible association with αE-catenin and plakophilins, suggested by our data, needs to be validated at the functional level. Since the full-length Armc8α was unable to interact in our Y2H approach with any of the interaction partners identified for truncated Armc8 variants, it is conceivable that ectopic full-length Armc8α exists in a closed confirmation in yeast, which lacks endogenous Armc8, and that for instance post-translational modifications are necessary to open up this structure to make the central interaction region in Armc8 accessible for αE-catenin or plakophilin binding in yeast. Another explanation for our observations might be that a yeast protein is associating with full-length Armc8, in this way hiding the interaction region in full-length but not truncated Armc8.

Interestingly, except for p0071, Armc8 did not interact with the other CTNND core members: p120-catenin, δ-catenin or ARVCF (Figure 1D). While all CTNND core members co-localize with classical cadherins at adherens junctions, plakophilins (encoded by *PKP1* to *-3*) show a more restricted localization in desmosomes as they interact with desmosomal cadherins and desmoplakin (reviewed in [37]). p0071 catenin (encoded by *PKP4*) is exceptional in the δ-catenin family, as it can play a role in both adherens junctions and desmosomal junctions [18,38]. Armc8 is involved in the negative regulation of adherens junctional CCC complex members [10] possibly through the degradation of αE-catenin [8]. The results of our study suggest that Armc8 might be involved as well in the regulation of desmosomal cell adhesion through its interaction with plakophilins and p0071. The exact functional role of Armc8 in desmosomal junctions remains, however, to be elucidated. Currently, the degradation of the desmosomal pool of the plakophilins and p0071 is poorly studied and Armc8 might be a strong candidate to be involved in this. There is mounting evidence that not only inactivation of the CCC complex but also this of desmosomes is involved in the progression of cancer [39]. In addition to their structural function, desmosomes also act as signaling platforms involved in the regulation of cell proliferation, differentiation, migration, morphogenesis and apoptosis [39]. Additional studies are necessary to explore the influence of Armc8 expression on desmosomes in human cancer.

## Supporting information

**Supplementary Figure S1:** 

**Supplementary Figure S2:** 

**Supplementary Table S1:** 

**Supplementary Table S2:** 

**Supplementary Table S3:** 

**Supplementary Table S4:** 

**Supplementary Table S5:** 

## Abbreviations

Armc8: armadillo-repeat-containing protein 8
CCC: E-cadherin/β-catenin/α-catenin
