# Supplementary material for: Armc8 is an evolutionarily conserved armadillo protein involved in cell–cell adhesion complexes through multiple molecular interactions
Source: Biosci Rep. 2019 Aug 2;39(8):BSR20180604. doi: 10.1042/BSR20180604 (PMC6680376; doi:10.1042/BSR20180604)
Supplement: Supplementary file 1 [file bsr20180604_Supp1.pdf]

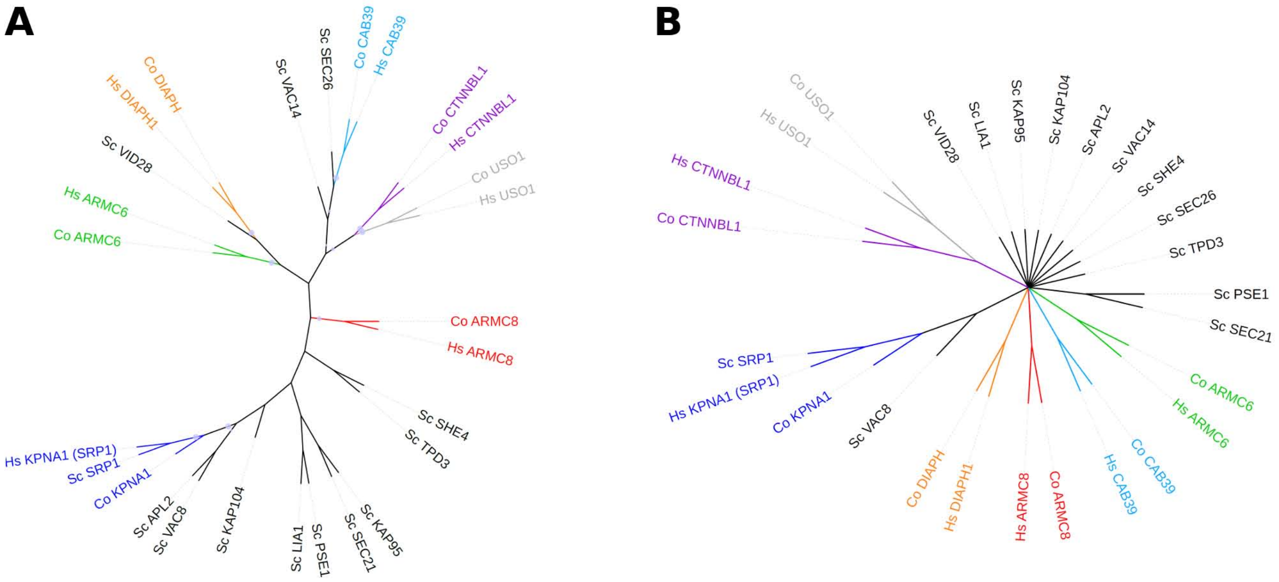
A
Co DIAPH
Hs DIAPH1
Sc VID28
Hs ARMC6
Co ARMC6
Sc VAC14
Sc SEC26
Co CAB39
Hs CAB39
Co CTNNBL1
Hs CTNNBL1
Co USO1
Hs USO1
Co ARMC8
Hs ARMC8
Sc SHE4
Sc TPD3
Hs KPNA1 (SRP1)
Sc SRP1
Co KPNA1
Sc APL2
Sc VAC8
Sc KAP104
Sc LIA1
Sc PSE1
Sc SEC21
Sc KAP95
B
Co USO1
Hs USO1
Hs CTNNBL1
Co CTNNBL1
Sc VID28
Sc LIA1
Sc KAP95
Sc KAP104
Sc APL2
Sc VAC14
Sc SHE4
Sc SEC26
Sc TPD3
Sc PSE1
Sc SEC21
Sc SRP1
Hs KPNA1 (SRP1)
Co KPNA1
Sc VAC8
Co DIAPH
Hs DIAPH1
Hs ARMC8
Co ARMC8
Hs CAB39
Co CAB39
Co ARMC6
Hs ARMC6

A

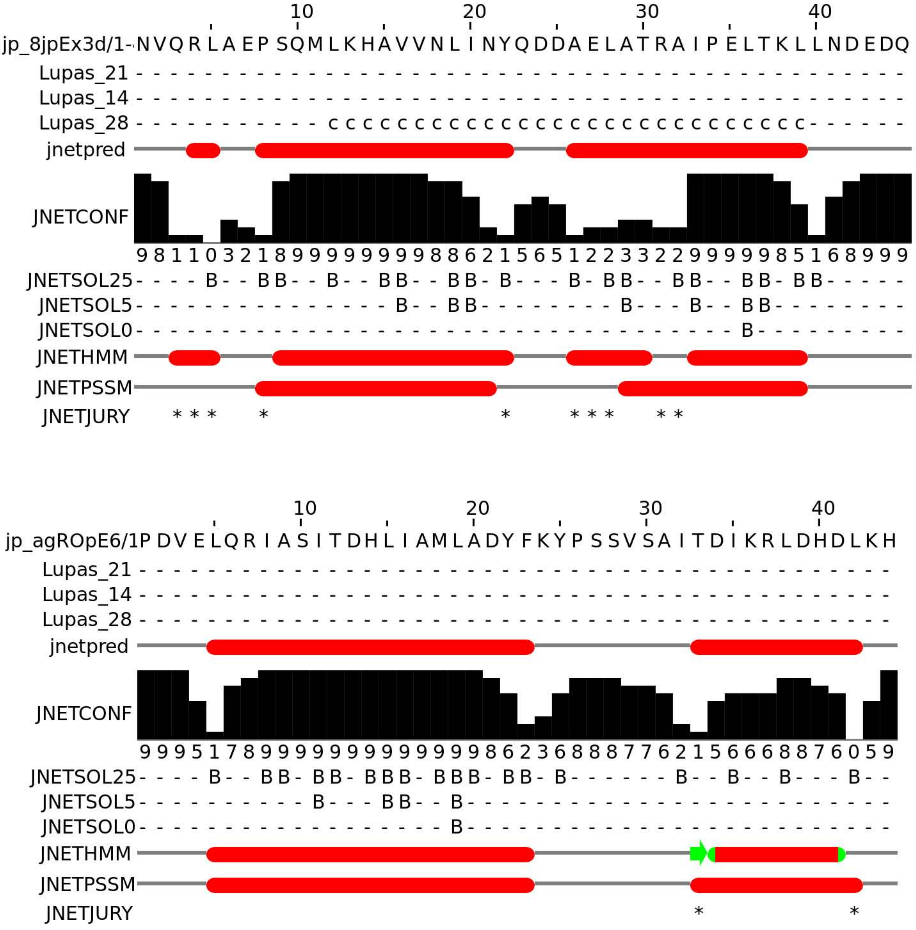

10 20 30 40
jp_8jpEx3d/1-·NVQRLAEPSQMLKHAVVNLINYQDDAELATRAIPELTKLLNDEDQ
Lupas_21 ---
Lupas_14 ---
Lupas_28 -----------cccccccccccccccccccccccccccc------
jnetpred
JNETCONF
981103218999999998862156512233229999985168999
JNETSOL25 ----B--BB--B--BB--BB-B---B-BB--BB--BB-BB-----
JNETSOL5 --------------B--BB--------B---B--BB--------
JNETSOL0 -----------------------------------B---------
JNETHMM
JNETPSSM
JNETJURY * * * * * * * * * *
10 20 30 40
jp_agROpE6/1PDVELQRIASITDHLIAMLADYFKYPSSVSAITDIKRLDHDLKH
Lupas_21 --
Lupas_14 --
Lupas_28 --
jnetpred
JNETCONF
99951789999999999998623688877621566688776059
JNETSOL25 ----B--BB-BB-BBB-BBB-BB-B------B--B--B---B--
JNETSOL5 ----------B---BB--B-------------------------
JNETSOL0 -----------------B--------------------------
JNETHMM
JNETPSSM
JNETJURY * *

B

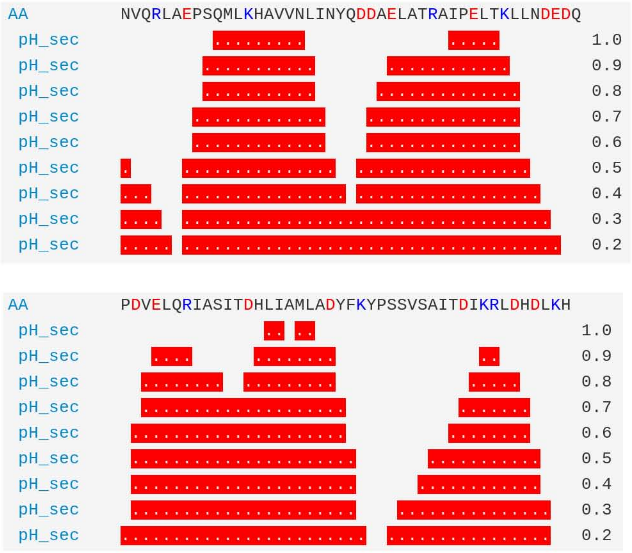

AA NVQRLAEPSQMLKHAVVNLINYQDDAELATRAIPELTKLLNDEDQ
pH_sec 1.0
pH_sec 0.9
pH_sec 0.8
pH_sec 0.7
pH_sec 0.6
pH_sec 0.5
pH_sec 0.4
pH_sec 0.3
pH_sec 0.2
AA PDVELQRIASITDHLIAMLADYFKYPSSVSAITDIKRLDHDLKH
pH_sec 1.0
pH_sec 0.9
pH_sec 0.8
pH_sec 0.7
pH_sec 0.6
pH_sec 0.5
pH_sec 0.4
pH_sec 0.3
pH_sec 0.2

	Construct	Forward	Reverse
1	pDONR207 hs Armc8α	5'-ATGGAAGTAACAGCTAGCAGTCG-3'	5'-TCATGCCAGGTACTGCTGCAG-3'
2	pDONR207 hs Armc8α(C-term)	5'-CCTTGTTTGGTTCGAATGTGCA-3'	5'-TCATGCCAGGTACTGCTGCAG-3'
3	pDONR207 hs Armc8α(2nd ARM)	5'-ATCATTGAGACTGAAAAT-3'	5'-TCATGCCAGGTACTGCTGCAG-3'
4	pDONR207 hs Armc8β	5'-ATGGAAGTAACAGCTAGCAGTCG-3'	5'-TCAGCAGGGAGTGACGTCAACC-3'
5	pDONR207 hs Armc8β(end)	5'-CCTTGTTTGGTTCGAATGTGCA-3'	5'-TCAGCAGGGAGTGACGTCAACC-3'

Construct	Cloning
pGBKT7 Pkp1	Described in [20]
pAS2 Pkp2	Described in [20]
pGBKT7 Pkp3	Described in [19]
pGBKT7 Pkp3(ARM + C-terminal)	Described in [19]
pGBKT7 Ctnna1	Described in [15]
pAS2 Ctnna2	Described in [17]
pGADT7 Ctnna3	Described in [15]
pGBKT7 Ctnna3	Described in [15]
pGBKT7 p120	Described in [19]
pGBKT7 Arvcf	Described in [18]
pGBKT7 Ctnna2	Described in [15]
pGBKT7 p0071	Described in [18]
pGBKT7 p0071 ΔC2	Described in [18]
pGBKT7 p0071 ΔN2	Described in [18]
pGBKT7 p0071 tail short	Described in [18]
pGADT7 Armc8α	LR reaction with #1 in Table S1
pdcDNA FLAG Armc8α	LR reaction with #1 in Table S1
pGADT7 Armc8α(pl)	LR reaction with #2 in Table S1
pGADT7 Armc8α(2nd ARM)	LR reaction with #3 in Table S1
pGADT7 Armc8β	LR reaction with #4 in Table S1
pGADT7 Armc8β end	LR reaction with #5 in Table S1

S. cerevisiae		***H. sapiens***			
**Symbol**	**Reference Sequence**	**Symbol**	**Description**	**E-value**	**Reference Sequence**
Gid1/Vid30	NP_011287.1	RANBP10	ran-binding protein 10	5.00E-16	NP_001307167.1
Gid2/Rmd5	NP_010541.3	RMND5A	protein RMD5 homolog A	2.00E-25	NP_073617.1
Gid4/Vid24	NP_009663.1	GID4	glucose-induced degradation protein 4 homolog	6.00E-22	NP_076957.3
Gid5/Vid28	NP_012247.3	TSPYL2	testis-specific Y-encoded-like protein 2	0.83	NP_071400.1
Gid7	NP_009891.1	WDR26	WD repeat-containing protein 26 isoform b	5.00E-14	NP_001108585.2
Gid8	NP_013854.1	---	No Significant Hits (E-value < 10)		
Gid9/Fyv10	NP_012169.1	MAEA	macrophage erythroblast attacher isoform 1	2.00E-13	NP_001017405.1

Armc8 Orthologs		*S. cerevisiae*		
Query		Description hit	E-value	Best hit
C. owczarzaki	XP_004345485	Atg26p	0.64	NP_013290.1
A. queenslandica	XP_003388104	No Significant Hits (E-value < 10)		
T. adhaerens	XP_002110308	No Significant Hits (E-value < 10)		
N. vetensis	XP_001637905	Kap123p	0.008	NP_011035.1
A. californica	XP_005111150	No Significant Hits (E-value < 10)		
B. floridae	XP_002598418	Kap123p	0.5	NP_011035.1
D. rerio	NP_001073151	Kap123p	0.018	NP_011035.1
G. gallus	NP_001239092	Kap123p	0.008	NP_011035.1
M. musculus	NP_083044	Kap123p	0.12	NP_011035.1
H. sapiens	NP_056211	Kap123p	0.13	NP_011035.1

SequenceID	Protein RefSeq	Length	GeneID	Source	Remarks
YEL013W	NP_010903.3	578 aa	VAC8	NCBI Gene; hmmsearch	ARM repeats
YNL287W	NP_014112.1	935 aa	SEC21	NCBI Gene	ARM repeats; HEAT repeats
YDR238C	NP_010524.3	973 aa	SEC26	NCBI Gene	ARM repeats
YOR035C	NP_014678.1	789 aa	SHE4	NCBI Gene	ARM repeats
YNL189W	NP_014210.1	542 aa	SRP1	superfamily; NCBI Gene; hmmsearch	ARM repeats
YBR017C	NP_009573.1	918 aa	KAP104	superfamily; hmmsearch	HEAT repeats
YLR347C	NP_013451.1	861 aa	KAP95	superfamily; hmmsearch	HEAT repeats
YAL016W	NP_009386.1	635 aa	TPD3	hmmsearch	HEAT repeats
YJR070C	NP_012604.1	325 aa	LIA1	hmmsearch	HEAT repeats
YKL135C	NP_012787.1	726 aa	APL2	hmmsearch	HEAT repeats
YLR386W	NP_013490.3	880 aa	VAC14	hmmsearch	HEAT repeats
YMR308C	NP_014039.1	1089 aa	PSE1	hmmsearch	HEAT repeats
YDL126C*	NP_010157.1	835 aa	CDC48	NCBI Gene	no ARM/HEAT repeats; only one TIP49 superfamily domain
YKL213C*	NP_012709.1	715 aa	Doa1p	NCBI Gene	no ARM/HEAT repeats; only WD40 repeats, PFU and PUL superfamily domain
YKL010C*	NP_012915.3	1483 aa	UFD4	NCBI Gene	no ARM/HEAT repeats; contains HUL4/HECTc domain
YGL238W*	NP_011276.1	960 aa	CSE1	superfamily	no ARM/HEAT repeats
YIL017C	NP_012247	921 aa	**VID28**	**not found by hmmsearch or supfam**	HEAT repeats
**NCBI Gene search:** armadillo AND "Saccharomyces" [orgn]					
**superfamily (supfam.org) search:** Armadillo repeat family				4 significant hits	
**hmmsearch** with Arm.hmm (PF00514) from Pfam (http://pfam.xfam.org)				9 significant hits	
* not included in phylogenetic analysis					
